# The Mammary Microenvironment in Mastitis in Humans, Dairy Ruminants, Rabbits and Rodents: A One Health Focus

**DOI:** 10.1007/s10911-018-9395-1

**Published:** 2018-04-28

**Authors:** Katherine Hughes, Christine J. Watson

**Affiliations:** 1 Department of Veterinary Medicine, University of Cambridge, Madingley Road, Cambridge, CB3 0ES UK; 2 Department of Pathology, University of Cambridge, Tennis Court Road, Cambridge, CB2 1QP UK

**Keywords:** Mammary gland, Mastitis, Microenvironment, One Health, Rabbit, Ruminant, Sheep

## Abstract

The One Health concept promotes integrated evaluation of human, animal, and environmental health questions to expedite advances benefiting all species. A recognition of the multi-species impact of mastitis as a painful condition with welfare implications leads us to suggest that mastitis is an ideal target for a One Health approach. In this review, we will evaluate the role of the mammary microenvironment in mastitis in humans, ruminants and rabbits, where appropriate also drawing on studies utilising laboratory animal models. We will examine subclinical mastitis, clinical lactational mastitis, and involution-associated, or dry period, mastitis, highlighting important anatomical and immunological species differences. We will synthesise knowledge gained across different species, comparing and contrasting disease presentation. Subclinical mastitis (SCM) is characterised by elevated Na/K ratio, and increased milk IL-8 concentrations. SCM affecting the breastfeeding mother may result in modulation of infant mucosal immune system development, whilst in ruminants notable milk production losses may ensue. In the case of clinical lactational mastitis, we will focus on mastitis caused by *Staphylococcus aureus* and *Escherichia coli*. Understanding of the pathogenesis of involution-associated mastitis requires characterization of the structural and molecular changes occurring during involution and we will review these changes across species. We speculate that milk accumulation may act as a nidus for infection, and that the involution ‘wound healing phenotype’ may render the tissue susceptible to bacterial infection. We will discuss the impact of concurrent pregnancy and a ‘parallel pregnancy and involution signature’ during bovine mammary involution.

## Introduction

In this review, we will evaluate the role of the mammary microenvironment in mastitis in humans, ruminants and rabbits, where appropriate also drawing on studies utilising laboratory animal models. We will examine subclinical mastitis, clinical lactational mastitis, and involution-associated, or dry period, mastitis, which is more common in ruminants than in humans. We will synthesise knowledge gained across different species, comparing and contrasting disease presentation between humans and other species. For clarity and precision, the term ‘breast’ will be used solely to refer to the human mammary gland in the ensuing discussion.

Breast pain is one of the two most common problems faced by breastfeeding women [1]. Mastitis affects up to 33% of lactating mothers and is a major cause of precocious weaning [2, 3], with the concomitant short- and long-term health consequences for the child [4]. In addition, it has recently been suggested that subclinical mastitis may influence the infant’s mucosal immune system [5]. Similarly, in dairy cows and sheep, both acute clinical disease and chronic subclinical mastitis cause significant pain [6, 7] and thus present a major welfare problem. Mastitis represents around a third of the direct costs of all common dairy diseases [8] and has public health repercussions, particularly associated with the increased use of antimicrobials for disease treatment. Mastitis is also a major health concern in breeding does on rabbit farms [9–11]. Indeed, mastitis has been recorded amongst the most common reasons for culling does in Spanish commercial rabbitries [12] with mastitis the reason for euthanasia of 33% of the females culled in one study [13]. In a separate study of rabbit farms in Spain and Portugal, the prevalence of mastitis was 4% in lactating does [14]. In addition, mastitis may occur in pet female rabbits that are lactating or that develop pseudopregnancy. Mammary gland trauma or poor hygiene may be predisposing factors in such cases [15].

One Health is a term embodying the notion that there is a complex interplay between human and veterinary medicine and the environment, all of which impact human and animal health [16]. This concept can be applied to specific problems in which human and animal health clearly intersect, such as zoonotic diseases, or the responsible usage of antimicrobials [17], but the One Health focus of biomedical research also extends beyond such specific issues to promote an integrated evaluation of human, animal, and environmental health questions in a manner conducive to expediting conceptual advances and meaningful solutions benefiting all species [18]. One Health thus offers recognition that both human and veterinary medicine can “contribute to the development of each other” [16] and, amongst many other topics, encompasses molecular and microbiology as they relate to comparative and translational medicine [17]. Given the multi-species impact of mastitis as a painful condition potentially influencing the welfare of both the mother and offspring, together with its wider economic and public health implications, we suggest that mastitis is an ideal target for a One Health approach.

**Fig. 1:**
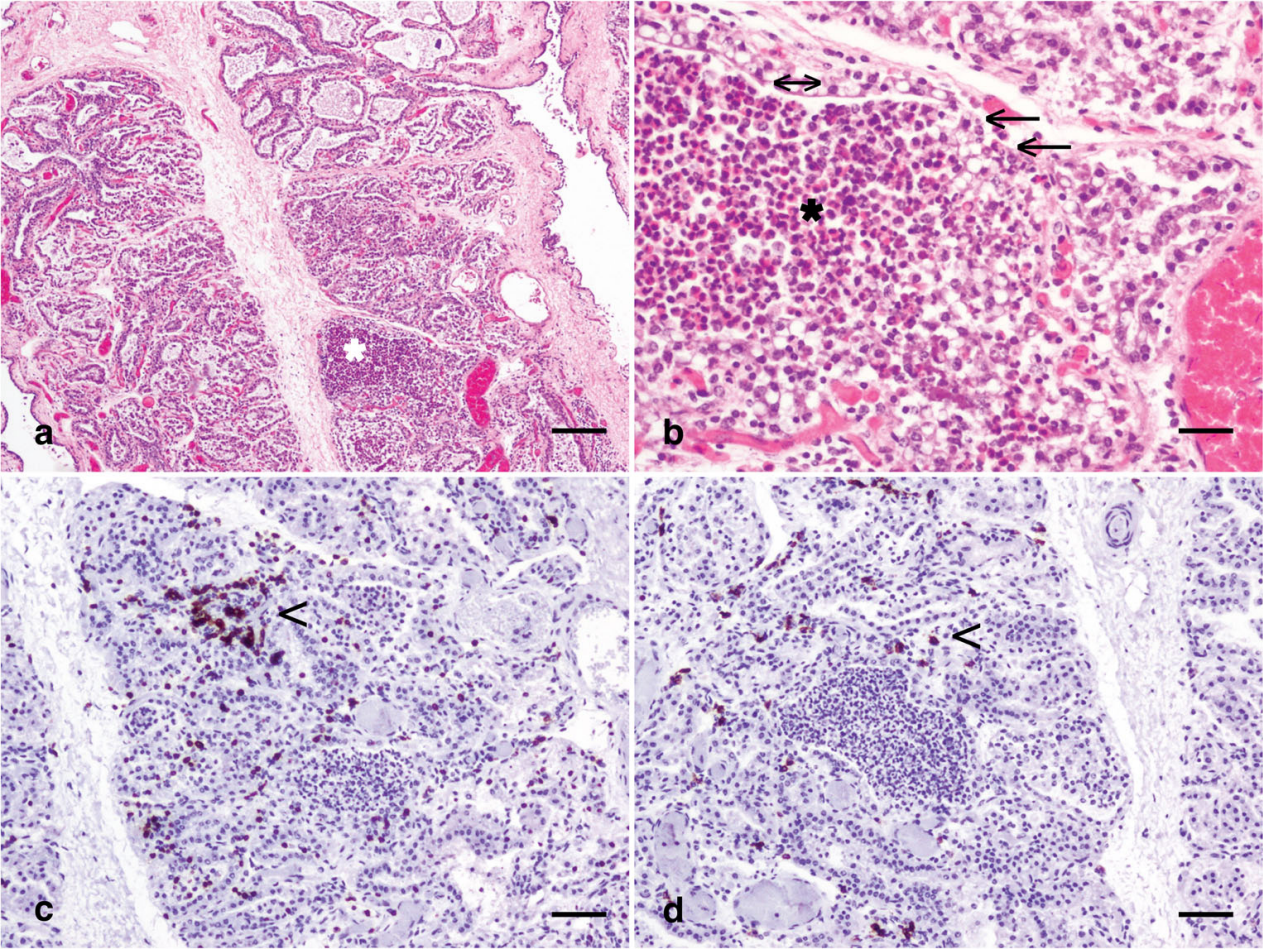
Th**e mammary microenvironment in mastitis in a third lactation Holstein Friesian cow, 46 dL.** (a) Multifocal mammary alveoli are engorged with numerous predominantly degenerate neutrophils (*). Haematoxylin and eosin stain; scale bar: 300 μm. (b) Severely affected alveoli with myriad neutrophils (*) exhibit partial loss of the luminal epithelial lining (arrows) although the partial remnants of the mammary epithelial lining remain (double headed arrow). Haematoxylin and eosin stain; scale bar: 50 μm. (c) Scattered aggregates of lymphocytes expressing CD3 (arrowhead) are present multifocally. Immunohistochemical staining for CD3 with haematoxylin counterstain; scale bar: 100 μm. (d) Rarer individual lymphocytes expressing CD20 (arrowhead) are present between mammary alveoli. Immunohistochemical staining for CD20 with haematoxylin counterstain; scale bar: 100 μm. dL: days lactation

The mammary microenvironment comprises luminal and basal epithelial cells, stromal components including fibroblasts, endothelial cells and stromal matrix, and the immune cell compartment. These mammary constituents engage in a complex and interweaving network of interactions, with physical connectivity, and paracrine and hormonal influences, orchestrating the interplay. Pathogen biology is frequently at the forefront of mastitis studies, but it is evident that the response of the mammary epithelium and the microenvironment to the pathogen is also critical (Fig. 1), and elements of the microenvironment may provide novel therapeutic targets [19].

## Comparative Mammary Gland Anatomy and Development

One challenge associated with consideration of mastitis from a One Health perspective revolves around an appreciation of species differences in anatomy which may impact the pathogenesis of mastitis. For example the sinuses present in the ruminant mammary gland have a particular role in storage of milk, and may provide a medium for bacterial growth, as well as being a site where immune cells transit into the milk. Thus, the following section will consider comparative mammary gland anatomy and development, with twofold aims to both emphasise structures that may have a role in mastitis pathogenesis and to highlight differences between species.

The anatomy of the mammary gland has been studied for centuries [20] and species variations have been well-documented (Table 1). In addition to the externally obvious variations in the number of mammae, the number of galactophorous, or lactiferous, ducts also varies, and this impacts the number of divisions per mamma. Terminology in this field is variable, with some authors preferring the term ‘gland’ to describe these divisions [21], whilst others prefer the terms ‘lobes’ [22], ‘sectors’ [25] or ‘ductal systems’ [23]. We will use the term ‘ductal system’ to refer to this division in the following discussion. Bovine, ovine, caprine and murine mammae have one ductal system per mamma [24], the rabbit has approximately six or seven [26] (authors’ submitted manuscript) and the human breast has 4–18 [22–24] (Table 1).

**Table 1:** Species differences in mammary anatomy [21]

Species	Number of pairs of mammae (mammary glands)				Ductal systems per mamma	References
	Cervical	Thoracic	Abdominal	Inguinal		
Human		1			4–18	[22–24]
Cow				2	1	[24]
Sheep and goats				1	1	[24]
Rabbit		1	2	1	Approximately 6	[25–27] Authors’ unpublished data
Mouse	1	2	1	1	1	[24]

**Fig. 2:**
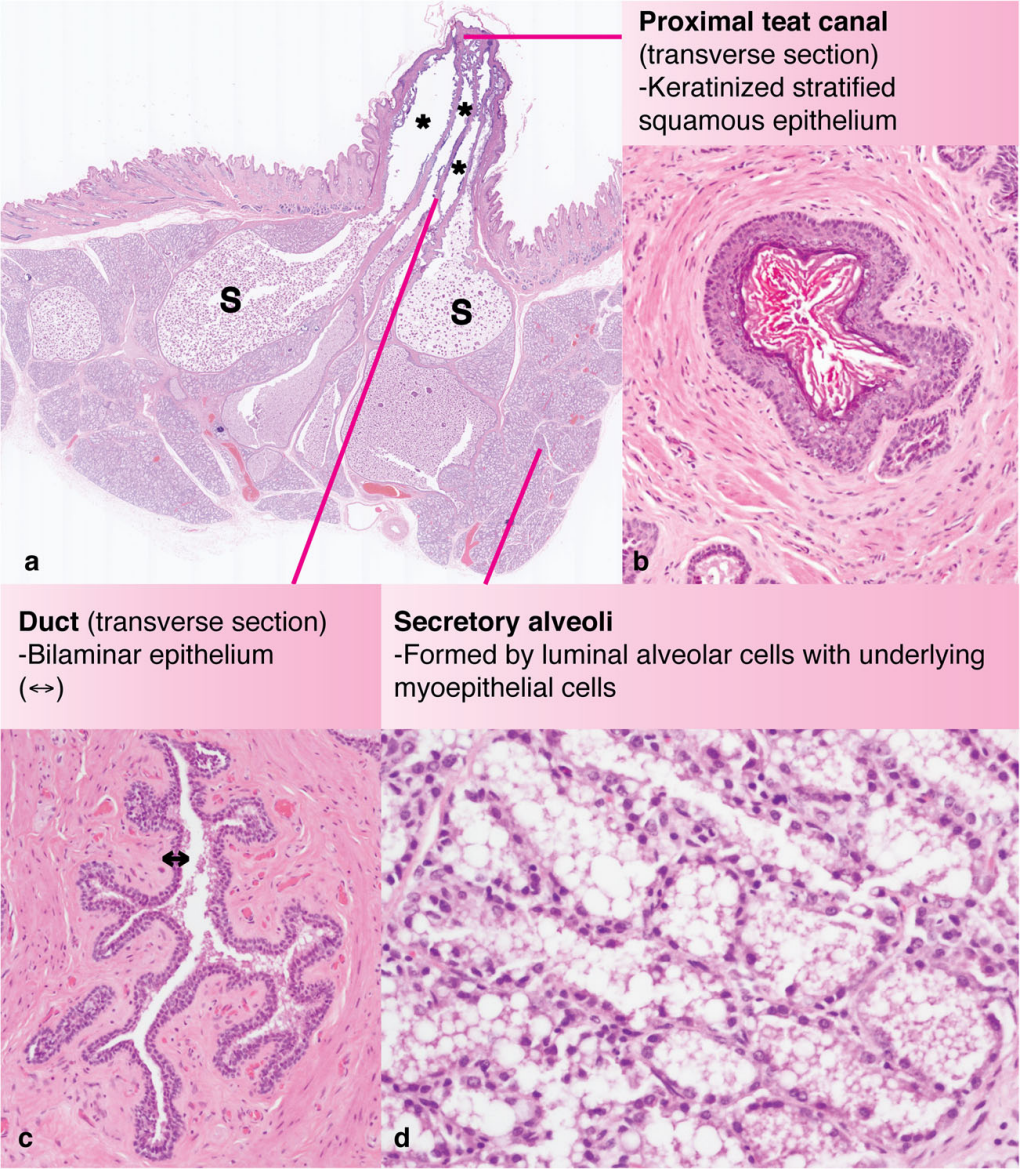
**Sub-gross anatomy and histology of the rabbit mammary gland.** (a) Sub-gross histological section (sagittal plane) through the teat and mammary tissue of a wild rabbit, *Oryctolagus cuniculus*, during late pregnancy, estimated 27 dG. Multiple ducts (*) are apparent and exhibit dilatations suggestive of sinusoidal structures (S). (b) Transverse section of a rabbit teat canal, < 1 mm from the teat orifice, demonstrating the keratinized stratified squamous epithelium. (c) Transverse section of a mammary duct demonstrating the bilaminar epithelial lining (double headed arrow). (d) Mammary alveoli formed by a luminal layer of mammary epithelial cells and an underlying layer of myoepithelium. Haematoxylin and eosin stain; dG: days gestation

The important anatomical difference in the number of ductal systems per mamma arises during development. At approximately embryonic day (E) 15.5 in the female mouse embryo, the mammary primordium transitions to the sprout stage and the distal aspect of the bulb elongates to penetrate the deeper mesenchyme, termed the secondary mammary mesenchyme, (synonym: fat pad precursor mesenchyme). Subsequent branching morphogenesis of the sprout produces a rudimentary branched ductal system [28]. Similarly, in the rabbit, between E17 and E23, the bulb’s spherical aspect becomes larger and elongates to enter a deeper zone of adipose-rich mesenchyme. However, by contrast to the mouse, at E26 in the rabbit, the bud commences division, with each resulting sprout giving rise to a primary milk canal that subsequently undergoes branching morphogenesis. Hence the rabbit (and human) mamma exhibit multiple mammary trees, each with a primary milk canal dividing the mamma into different ductal systems [29, 30] (Fig. 2).

Comprehensive descriptions of mammary gland development in the human [31, 32], mouse [28–30], ruminant [24] and rabbit [30] already exist and this subject will only be discussed further in this review with respect to aspects of postnatal development, in particular post-lactational regression, which are relevant to the pathogenesis of mastitis arising in association with the ruminant dry period. In dairy cows, the dry period, particularly the time immediately following the end of the prior lactation and the time immediately preceding parturition, represents a phase of the mammary cycle when new intramammary infections may be acquired [33].

**Fig. 3:**
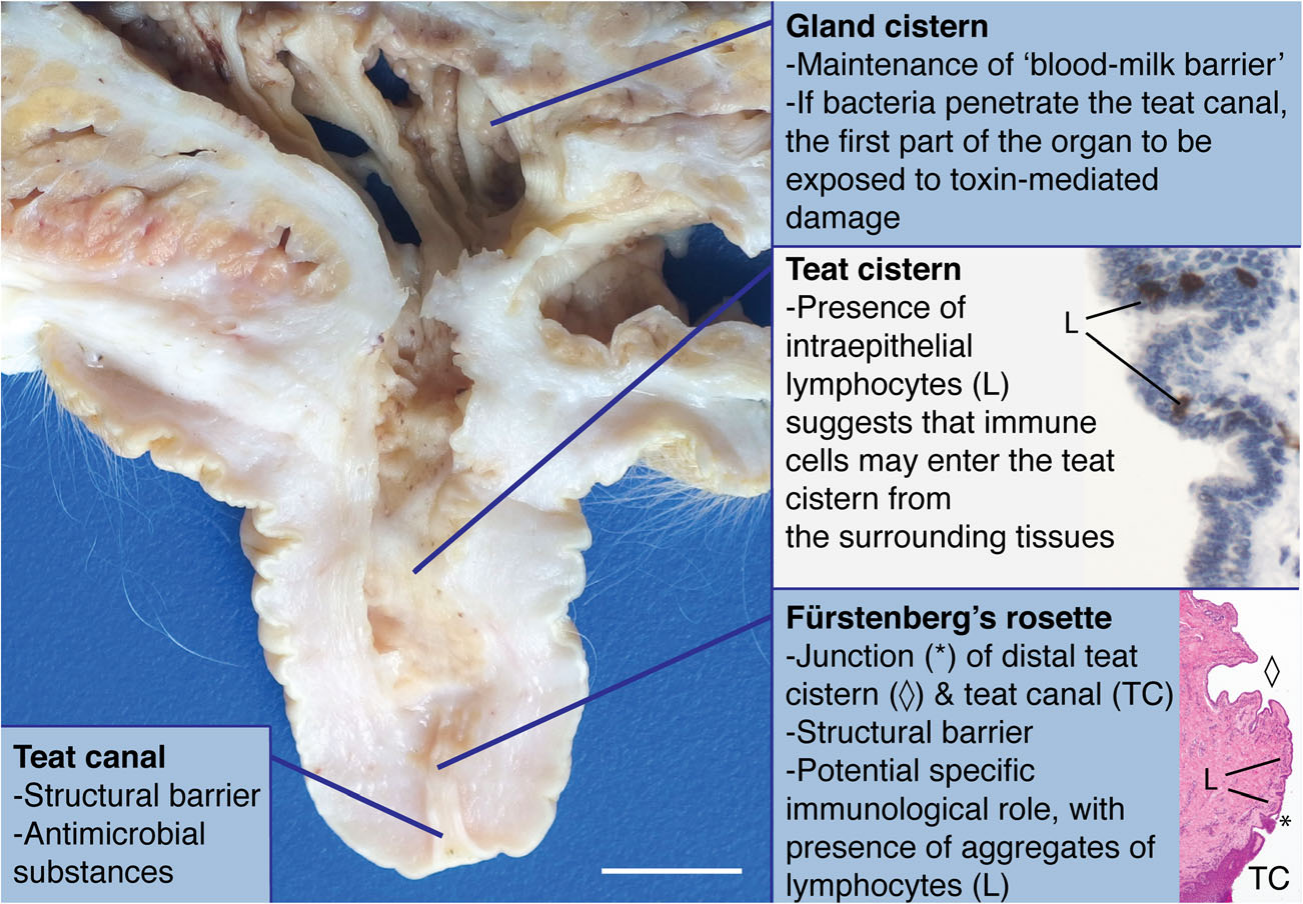
**Structural defences of the bovine mammary gland.** Sagittal section through the distal gland cistern, teat cistern and teat canal of a periparturient Holstein Friesian dairy cow; formalin fixed tissue; scale bar: 10 mm. Teat cistern immunohistochemical inset: Sagittal section through the teat cistern. Scattered intraepithelial lymphocytes expressing CD3 are present multifocally. Immunohistochemical staining for CD3 with haematoxylin counterstain. Fürstenberg’s rosette histological inset: Sagittal section through the teat canal (TC) – distal teat cistern (diamond) junction, Fürstenberg’s rosette (*) of a Holstein Friesian dairy cow 45 dI. Groupings of small to moderate numbers of lymphocytes (L) are present multifocally. Haematoxylin and eosin stain; dI: days involution, with concurrent pregnancy until abortion at approximately 31dI

In ruminants, the larger ducts from each mamma open into the gland cistern, which in turn communicates with the teat cistern that, itself, is connected with the exterior via the teat canal [25, 34] (Fig. 3). Early dissections describe the ductal anatomy of the lactating breast [20]. More recent studies have included three dimensional reconstruction from serial histological sections allowing the appreciation of ductal anatomy in three dimensions [35]. Magnetic resonance imaging has also been employed to delineate changes in breast morphology between lactation and weaning [36]. Although earlier work has depicted lactiferous sinuses within the breast [25], an ultrasound study of 21 lactating women demonstrated a frequent increase in duct diameter at multiple branch points, but an absence of lactiferous sinuses under the areola, leading the authors of that study to conclude that in the human breast, ducts act as a milk conduit rather than a storage sinus. Importantly, these authors also noted that the ducts were easily compressed [22]. Lactiferous sinuses have not been described in the mammary gland of the rabbit [25] and it has been suggested that milk is stored in the secretory alveoli [26] although we observe sinus-like dilatations of the milk ducts in the mammary gland of pregnant and lactating rabbits (Fig. 2) (submitted manuscript).

**Fig. 4:**
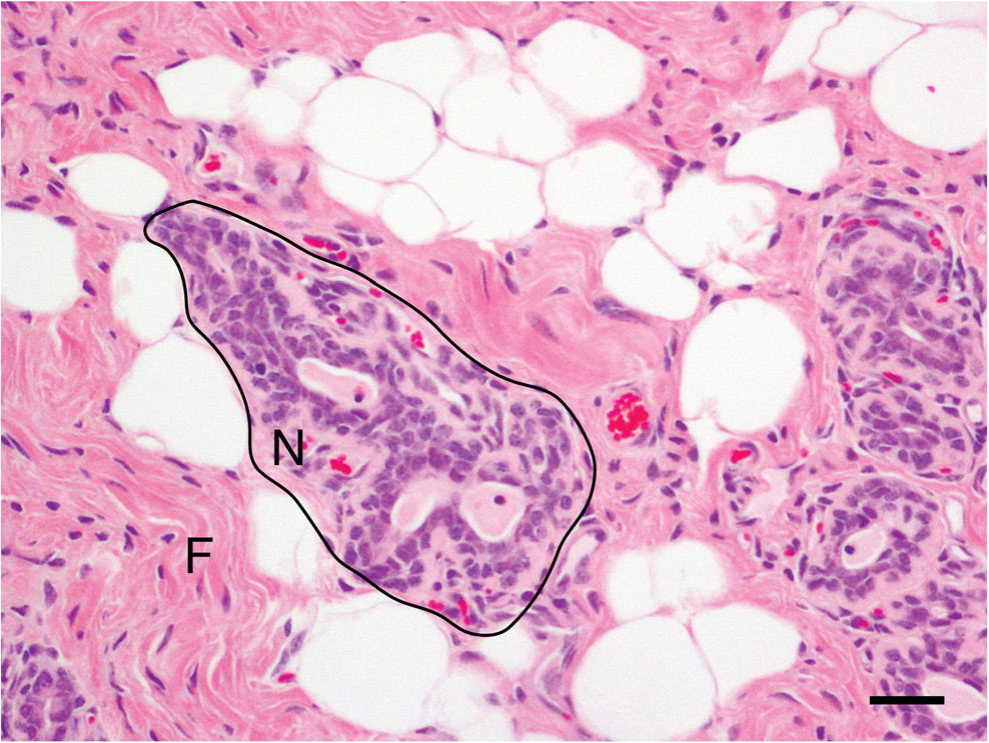
**Near- and far- stromal regions of the immature ovine mammary gland.** Histolological section from the mammary gland of a nulliparous 18-month-old ewe lamb. Near-stromal regions (N) are within approximately 75 μm of mammary epithelial structures. Far-stromal regions (F) comprise both collagen rich connective tissue and adipose. Haematoxylin and eosin stain; scale bar: 50 μm

The mammary stroma also varies between species. In rodents, the mammary fat pad is predominantly composed of adipocytes. In the lactating breast, the stroma is also adipose-rich [22], whilst in the ruminant, the stroma comprises adipose dissected by trabeculae of collagen [25, 37]. Interestingly, in the developing bovine mammary gland, authors recognise ‘far stromal’ regions comprising a combination of adipose and fibrous tissue, and more dense ‘near stromal’ tissue regions which are arranged within a 100 to 150 μm radius of mammary epithelial structures [38]. We have observed similar ‘far stromal’ and ‘near stromal’ zones in the mammary gland of ewe lambs (Fig. 4).

## Comparative Mammary Gland Immunology

It is not the purpose of this review to provide a comprehensive account of immunology of the mammary gland, and, indeed, other authors have provided detailed insights into this subject in humans and mice [39, 40] and dairy ruminants [41, 42]. However, it is pertinent to mention several aspects of comparative mammary gland immunology which are particularly relevant to the study of mastitis from a One Health perspective.

### Teat Sphincter, Teat Canal, Fürstenberg’s Rosette, and Teat Cistern

Several elements of the ruminant mammary immune defences derive from the particular anatomy of the udder as described above [42]. The teat sphincter is an important anatomical structure maintaining closure of the teat canal [43, 44]. Recently, cells situated near smooth muscle cells and exhibiting cytoplasmic processes, and dual positivity for CD117 and vimentin, have been identified and suggested to represent bovine telocytes. It has been postulated that these cells may have a role in regulating the contractility of the sphincter and thus may act as a component of the innate immune system of the ruminant mammary gland [45].

Located at the proximal end of the bovine teat canal (Fig. 3), the Fürstenberg’s rosette (FR) is a small region between the streak canal and the teat cistern which has 4–18 folds and is 2–11 mm in width, [46]. It is not clear whether the FR solely forms an integral component of the physical barrier of the teat or whether it has a more specific immunological role. However, at the mRNA expression level, the FR exhibits higher constitutive expression of S100 proteins (A8, A9, A12) than the teat cistern, perhaps implying a distinct immunological function [47]. Intriguingly, the FR is described as having a protective leukocyte population, and the presence of intraepithelial leukocytes suggests that some of these immune cells leave the teat wall and enter the cistern [48] (Fig. 3).

In a large histological study of ovine teats from clinically healthy animals, lymphocytes were the predominant immune cell type in the teat cistern, whereas in the teat duct, similar numbers of lymphocytes and neutrophils were observed. Subepithelial lymphoid nodules, most frequently at the border between teat duct and teat cistern, were detected in 49% of the samples examined, and were significantly associated with the presence of bacteria [49]. Thus, in sheep, it also seems likely that the presence of subepithelial lymphoid tissue at the border between teat duct and teat cistern is an important component of mammary defence but to fully elucidate the role of these aggregates it is critical to distinguish between pre-existing lymphoid accumulations and those induced by bacterial invasion [49–51]. This is an example of an area in which a One Health approach may offer useful insights for future research and where the relevance of findings derived from one species is yet to be tested in other species. For example, we observe lymphocytes at the junction between the teat canal and the mammary duct in rabbits (authors’ unpublished data) but, as yet, the significance of these groupings of lymphocytes remains undefined.

### The Mammary Epithelial Cell as A Component Of The Mammary Immune System

It is well-established that mammary epithelial cells themselves are important players in the mammary immune microenvironment. We have previously demonstrated that, during murine post-lactational regression, mammary epithelial cells exhibit dramatic, Stat3-dependent, up-regulation of CD14, an innate immune component [52, 53]. In addition, cultured bovine and caprine mammary epithelial cells have been shown to express TLR2 and TLR4 mRNA, and responses of mammary epithelial cells to stimulation with bacterial cell wall components, or other agents with the potential to cause mastitis, such as *Prototheca* spp., result in elaboration of a plethora of cytokines and other inflammatory mediators [54, 55]. These findings suggest that mammary epithelial cells, across species, have a critical role to play in the mammary immune microenvironment although clearly much of the current evidence for this assertion is derived from studies using rodent mammary epithelial cells *in vitro,* and from *in vivo* studies of rodent models.

Mammary epithelial cells are also recognised to have phagocytic capability, perhaps best illustrated by work demonstrating the role of mammary epithelial cells in the process of efferocytosis. Efferocytosis comprises the phagocytic removal of superfluous or damaged cells by either neighbouring tissue cells or professional phagocytes [56] and is particularly important during mammary gland involution, the process by which the glandular architecture is remodelled at the cessation of lactation. It has been demonstrated that murine mammary epithelial cells are important effectors of efferocytosis during involution, and that this process is dependent on the autophagy related proteins Becn1 and ATG7 [57] and the receptor tyrosine kinase MerTK [58]. Interestingly, the study of the mammary gland of sheep has also yielded morphological evidence of epithelial cell efferocytosis during mammary gland involution [59]. Mammary epithelial cells also use phagocytosis to take up large milk fat globules accumulating in the mammary alveolar lumen during early involution [60], further underlining the ability of the glandular epithelial cells to acquire a phagocytic phenotype.

### Soluble Components of Mammary Gland Immunity

There are a number of soluble proteins that constitute important components of mammary gland immunity, including lactoferrin, transferrin, the lactoperoxidase and myeloperoxidase systems, complement proteins, and lysozyme [42]. However, drawing comparisons across species necessitates an awareness of species variations in the synthesis of such components, and the concentrations of these components in milk. For example, lysozyme is a bactericidal protein present in milk which catalyses the hydrolysis of peptidoglycan residues in bacterial cell walls. Lysozyme appears to be an important immune component of human milk but is present at a much lower concentration in bovine milk [61].

## Subclinical Mastitis

The term subclinical mastitis (SCM) is used to indicate inflammation of the breast or mamma which does not result in clinically detectable symptoms. A notable condition in both humans and milk production animals, SCM is associated with decreased milk quality and yield in dairy ruminants [62] and presumably a similar phenomenon in humans, where it has been associated with reduced infant weight gain [63]. Importantly, it is increasingly recognised that breast milk consumption may influence the immunological development of babies [64, 65] and that SCM may therefore impact this development [5]. In addition, SCM has particularly significant potential sequelae for infants breast fed by women with lentiviral infections, and this topic will be discussed further at the end of this section.

In humans, diagnosis of SCM is reached when there is an elevated milk sodium/potassium (Na/K) ratio above 1.0, and an increased concentration of interleukin-8 (IL-8), in the absence of clinical signs [66]. In dairy ruminants, milk somatic cell count (SCC) or microbial culture-based methods are mainstays of diagnosis [67].

The study of SCM is particularly relevant to a One Health approach as human SCM is relatively poorly characterised, whereas bovine SCM, in particular, is a well-established research focus due to the high proportion of mastitis-associated losses attributed to SCM. Therefore it is possible, that testing insights from bovine SCM for their applicability to human SCM may be a fruitful avenue of future research in the One Health field. However, any such approach would require an outlook mindful of species differences as discussed elsewhere in this review.

### Changes in Milk Na/K Ratio During SCM

Fluctuations in the mammary microenvironment in human SCM are relatively poorly understood and alterations in milk components provide the main ‘window’ into understanding the changes which may be occurring in the breast at this time. As already mentioned, the milk Na/K ratio is perturbed during an episode of SCM, providing a diagnostic tool. To understand this phenomenon, it is necessary to consider changes occurring at the level of both the whole mammary gland, and individual mammary epithelial cells.

Once lactation has been established (at approximately day four post partum) breast milk Na/K ratio is primarily affected by extent of milk extraction from the gland. When demand for milk is reduced, which could be due to factors including breast feeding technique or supplementary feeding, the gland becomes distended. This potentially exacerbates reduced emptying, as the infant’s ability to suckle from an engorged breast may be compromised or the breast may be more prone to injury [68]. When considering breast engorgement as a factor in the development of subclinical mastitis, it is important to note that, as already described, in humans, milk ducts act as a milk channel rather than a storage sinus, and are readily compressible [22]. Furthermore, the processes described above can also contribute to a ‘vicious cycle’ where mastitis itself can contribute to compromised milk removal and breast engorgement [69].

The secretory activation phase of differentiation (formerly termed lactogenesis stage 2), prompted by progesterone withdrawal, commences at the termination of pregnancy in rodents and ruminants and shortly after parturition in humans, when the placenta is delivered [70, 71]. Importantly, secretory activation heralds the closure of tight junctions (synonym: zona occludins) between mammary epithelial cells [72]. However, if demand for milk declines, the mammary acini are progressively distended, the tight junctions between epithelial cells open and sodium influx into the milk increases. Leaky tight junctions, the consequent unrestricted movement of components of the interstitial space into the milk, and thus an elevated milk Na/K ratio, are important features of subclinical mastitis [73]. Although the gland will tend to down-regulate milk production to match demand, milk stasis may potentially lead to infection [68]. Buffaloes with SCM diagnosed on the basis of bacteriology and SCC also exhibit elevated milk Na and decreased K [74], further indicating that a perturbed Na/K ratio may be a feature of SCM irrespective of species.

In parallel with an increased Na/K ratio, milk concentrations of IL-8 increase in human SCM [75–77]. Interestingly, using quantitative real time polymerase chain reaction, expression of the IL-8 receptor has also been demonstrated to be significantly higher in milk somatic cells from crossbred dairy cows diagnosed with SCM, detected on the basis of the California Mastitis Test, SCC, and electrical conductivity test, when compared to healthy cows [78]. A recent study has demonstrated that human SCM, as defined by an Na/K ratio in excess of 1, is associated with higher levels of a panel of inflammatory mediators and markers, again including IL-8, but also including β2 microgobulin, PS100A9, TNF-α, IL-6, IL-17, RANTES, IL-2R, IL-12p40/70, IFN-α, IFN-γ, CXCL-9, IP-10, MIP-1α, MIP-1β, LPS binding protein, α-defensins, and antileukoproteinase 1 [5]. Again, evidence of a similar inflammatory profile can be inferred in dairy ruminants, where higher levels of TNF-α and IL-12 have been observed in ewes with SCM [79].

SCM has been implicated as a potential risk factor which increases the likelihood of mother-to-child Human Immunodeficiency Virus (HIV) transmission via breast milk [66]. *Staphylococcus aureus* is a frequently implicated pathogen in HIV-infected women in Malawi [80, 81]. The small ruminant lentiviruses (SRLVs) caprine arthritis and encephalitis virus (CAEV), maedi-visna virus (MVV), and ovine progressive pneumonia virus (OPPV) may also be shed in colostrum and milk [82]. Whilst it is well-established that SRLVs may cause mastitis, to the authors’ knowledge, it is not known specifically whether the presence of SCM increases the likelihood of SRLV vertical transmission.

## Clinical Lactational Mastitis

The normal bacterial content of human breast milk is increasingly recognised as critical to infant immune development [83, 84] and thus lactational mastitis presents particular challenges in human subjects where inappropriate antibiotic therapy for patients with lactational mastitis is likely to have profound effects on the microbiome [65]. Interestingly, in humans, the microbiome of mastitic milk has been demonstrated to have a reduced bacterial diversity [85, 86]. In veterinary medicine, much of the focus of mastitis research has centred upon the pathogenic organisms involved, which has led to a better appreciation of the epidemiology and pathogenesis of mastitis in the context of different pathogens. In 2014 a new paradigm for mastitis was proposed, emphasising the importance of host inflammatory mediators as drivers of disease [19]. We suggest that both approaches are relevant to the study of mastitis with a One Health focus and our discussion of lactational mastitis will focus on the microenvironmental response to two key mastitis pathogens. It is pertinent to emphasise here that taking a ‘pathogen approach’ to consideration of microenvironmental changes is not advocating use of antibiotic therapy in cases of human lactational mastitis.

### *Staphylococcus aureus*

In one human study, *Staphylococcus aureus* was the most frequently isolated pathogenic bacterium cultured from the breast milk of women with lactational mastitis [87] although the results of such studies must be interpreted with caution as other authors have identified the presence of potentially pathogenic bacteria in the breast milk of healthy women [88]. Bovine and ovine mastitis arising due to *S. aureus* infection may vary in severity, with a highly pathogenic, necrotizing, or gangrenous, form the most severe manifestation. Many cows also exhibit chronic mastitis or subclinical mastitis as a result of *S. aureus* infection [21].

Rabbits are also susceptible to *S. aureus* mastitis. This may present as a spectrum of lesions ranging from acute and necrotizing (gangrenous) forms [89], to more commonly chronic, suppurative, lesions [9, 11, 13] with variable levels of encapsulation [10]. In rabbits, the primary granulocytic leukocyte is frequently termed the heterophil rather than the neutrophil [27]. The diameter of a heterophil is 7–10 μm, the nucleus frequently exhibits multiple lobes and the cytoplasm exhibits eosinophilic granules [90]. Following lymphocytes, heterophils are the second most frequently encountered leucocyte in the rabbit, and comprise 20–75% of white blood cells [90]. Thus, purulent lesions in this species are microscopically characterised by accumulations of viable and degenerate heterophils [11]. Lesion cellularity in staphylococcal mastitis in rabbits may reflect mastitis chronicity, with greater numbers of T lymphocytes, macrophages and plasma cells in immature lesions [10].

In chronic *S. aureus* infections in bovine tissue, more IgG1- and IgG2- secreting leukocytes were detected at the FR when compared to uninfected quarters [91]. Experimental bovine udder challenge with *S. aureus* isolate 1027 (subclinical mastitis) has shown that there is an immune response by one hour after pathogen challenge, with up-regulation of expression of CCL20, CXCL8, TNF and IL-6 in the teat cistern [92]. In a longer time course udder inoculation model using another subclinical mastitis-causing strain of *S. aureus* (NCTC13047), IL-6, IL-17A, IL-8, and IL-10 were induced in the alveoli, ducts, gland cistern and teat canal of the bovine mammary gland. Expression of the acute phase proteins serum amyloid A3 and haptoglobin was induced, together with antimicrobial peptides [93]. Other investigators have demonstrated that intramammary challenge with lipoteichoic acid (LTA) from *S. aureus* will also increase mRNA expression of various cytokines including TNF-α, IL-1β, IL-8 and RANTES, and decreased lactoferrrin expression [94]. It has been suggested that the transcriptional changes documented in the bovine udder following challenge with *S. aureus* most likely echo the dual contributions of mammary epithelial cell activation and immune cell influx to changing gene expression profiles [95]. It has also been demonstrated that *S. aureus* can modulate Rho GTPase regulated pathways. Using actin fibres stained with fluorescent-labelled phalloidin, the same investigators have shown that primary bovine mammary epithelial cells cultured with *S. aureus* have an altered, more filamentous, actin cytoskeleton, which may facilitate bacterial invasion [96].

Excitingly, a recent study has demonstrated that, in a murine model, preconditioning the mammary gland with inoculation of LTA or lipopolysaccharide (LPS) modulates the innate immune response to a local *S. aureus* infection, and reduces the subsequent bacterial burden. By depleting macrophages in this model, the authors showed that this response was partially independent of macrophage signalling and the authors also implicated lipocalin 2 and chitinase 3-like 1 as potential modulators of the innate immune response [97].

Fibroblasts have an important role in chronic *S. aureus* mastitis as they facilitate collagen deposition and may mediate extensive glandular fibrosis. It has been suggested that transforming growth factor beta-1 may enhance *S. aureus* adhesion to, and invasion of, bovine mammary fibroblasts and that this interaction may be reduced using ERK inhibitors [98].

### *Escherichia coli*

In the cow, severe, necrotising, coliform mastitis may result from infection, with cows frequently also presented with concurrent endotoxaemia. Milder disease forms have also been reported [21]. Experimental intramammary administration of bacterial endotoxin results in the sloughing of large numbers of epithelial cells into milk [99]. In the same series of experimental bovine udder challenges as already described for *S. aureus*, challenge with *E. coli* resulted in enhanced up-regulation of expression of a wider panel of immune factors than *S. aureus*, with increases seen in CCL20, CXCL8, TNF, IL-6, IL-12b, IL-10, LAP and S100A9. However, the *E. coli* isolate used was a clinical mastitis isolate (1303) whereas the *S. aureus* strain was an isolate from a subclinical infection [92]. Intramammary challenge with LPS also shows stronger induction of expression of TNF-α, IL-1β, IL-8 and RANTES than LTA challenge, and similar decreased lactoferrrin expression. LPS challenge elicits increased concentrations of TNF-α in milk [94].

A microarray study in which cows received experimental intramammary administration of *E. coli* four to six weeks after calving has demonstrated that, in total, 982 transcripts are differentially expressed during the bovine host response to *E. coli* mastitis [100], with a wide network of pathways affected including complement and coagulation cascades, Jak-STAT signalling pathways and the toll-like receptor (TLR) signalling pathway (reviewed by [101]). The importance of TLR signalling is underlined by a study using TLR4 null mutant mice in which induction of mastitis via introduction of LPS resulted in initial higher abundance of neutrophils and macrophages but a reduced lesion infiltration of the same inflammatory cell types at 7 days post inoculation. Serum concentrations of certain cytokines, including CXCL1, CCL2, IL-1β, and TNF-α, were also reduced compared to wild-type mice [102]. However, following resolution of mastitis, milk production capacity was reduced in wild-type mice compared to those deficient in TLR4, raising the possibility that mastitis-associated lactation insufficiency may be due in part to TLR4-mediated inflammation, rather than bacterial infection *per se* [102].

## Involution- or Dry Period-Associated Mastitis

Mammary gland involution comprises the remodelling of the gland at the end of the lactation period. In humans, mastitis may potentially be associated with weaning particularly if the process is insufficiently gradual and the breast becomes engorged, with the pathogenesis potentially similar to that described to account for the elevated Na/K ratio used as a diagnostic modality in subclinical mastitis.

In ruminants, the start of involution, or the dry period, is well-recognised as a time when there is an increased likelihood of acquisition of new mammary infections, especially in cows with high milk yields prior to drying off [103]. ‘Summer mastitis’ is mastitis of dairy cows occurring during the summer months and therefore, in traditional systems, is associated with the dry period. This condition is usually caused by mixed bacterial species including *Trueperella pyogenes* and *Streptococcus dysgalactiae*. The route of infection is thought to be the papillary ostium and duct, and flies attracted to pre-existing teat lesions are implicated in the pathogenesis of the disease. In traditional systems, some of the affected animals at pasture would be dry, whilst others might be immature heifers [21].

In dairy small ruminants, it is suggested that pre-existing intramammary infections may recrudesce during the dry period and thus result in clinical disease. It is also documented that ewes/does are particularly susceptible to new infections [104]. In addition, in specific instances, a high incidence of mastitis is rarely seen at drying off in association with specific pathogens such as *Pseudomonas aeruginosa* or fungal agents [105].

Given the dramatic molecular and structural changes which occur in the mammary gland during post-lactational remodelling, we suggest that when considering episodes of dry period- or involution-associated mastitis, it may be informative to consider *changes occurring in the mammary microenvironment during involution* and to speculate on how these changes may influence *susceptibility* of the gland to mastitis-causing pathogens at this time. We will adopt this approach in the following section.

### Species Differences in the Mammary Microenvironment During Involution

Excitingly, a recent study used magnetic resonance imaging to describe changes seen in the breast during the first year post-weaning, and the investigators noted that the gland returns to a state similar to that observed pre-conception. Both breast area and fibroglandular fraction decreased significantly between women in the lactation and post weaning periods and measurements for the latter group were comparable to those of a premenopausal control group [36]. Such a striking degree of glandular remodelling is the result of highly regulated, interconnecting, networks of cellular signals which orchestrate the involution process [106]. Set at the hub of this molecular system is the transcription factor Signal Transducer and Activation of Transcription 3 (STAT3) [107, 108]. STAT3 activation is fundamental to the normal progression of involution [60, 109–111].

Mammary gland involution is considered to progress in two distinct phases, which have been well-defined in the mouse. The first reversible phase is proteinase independent and is characterised by dramatic mammary epithelial cell death co-ordinated by Stat3 [60, 109–111]. Mice with a mammary-specific conditional deletion of Stat3 exhibit a pronounced retardation of involution [109, 110]. Unilateral teat sealing, in which mice have sealant unilaterally applied to the inguinal mammary gland teat, such that pups sucking the contralateral open gland provide a continued systemic suckling stimulus, has demonstrated that local factors, attributed to milk accumulation, are sufficient to induce phosphorylation of Stat3 and consequent cell death at the onset of involution [112]. Leukemia inhibitory factor (LIF) exhibits a rapid increase in expression, which is independent of systemic factors, and is accordingly observed even in teat-sealed glands [113]. LIF deficient mice exhibit delayed mammary regression and absence of Stat3 activation during involution [114], demonstrating that the initial activator for Stat3 in the mammary gland is LIF, and that the up-regulation of pStat3 at the onset of involution is independent of the decrease in circulating lactogenic hormones seen after weaning. However, those glands which are sealed with the contralateral gland left open (thus maintaining systemic hormone stimulation) do not progress to the second phase of involution [112]. Thus exogenous administration of hydrocortisone, or systemic factors such as endogenous glucocorticoid release, can inhibit progression to the second phase [115].

When progression of involution is unimpeded, the second phase is accompanied by irreversible degradation of the mammary basement membrane, coinciding with expression, by fibroblasts and other mesenchymal components, of the matrix metalloproteinases (MMPs) MMP2 (gelatinase A), and MMP3 (stromelysin 1), the serine proteinase urokinase-type plasminogen activator [115], and MMP9 [53].

During the early phase of involution in mice, there is dramatic up-regulation of genes associated with the acute phase response and innate immunity, including serum amyloid A3 [52]. Stat3 regulates expression of a subset of these genes, including orosomucoids 1 and 2, secretory leukocyte protease inhibitor, CD14 and leucine-rich α2-glycoprotein 1 [53].

The later stages of involution are characterised by the mammary microenvironment acquiring an immunomodulatory ‘wound healing’ phenotype [116–118], which we have also demonstrated is dependent on Stat3 [53]. Factors implicated in acquisition of a ‘wound healing’ phenotype include deposition of fibrillar collagen, high levels of COX-2 expression, itself promoting lymphangiogenesis, and mammary epithelial cell efferocytosis [58, 119–121]. There is an influx of immune cells during the second phase of involution, including mast cells, lymphocytes, and predominantly alternatively activated macrophages [52, 53, 116, 122–124], and postlactational human breast tissue exhibits a transient infiltrate of high IL-10 (+) macrophages and Foxp3 (+) regulatory T cells [124]. It is easy to speculate that such an immunomodulatory and ‘wound healing’ microenvironment may favour proliferation of bacteria during involution-associated mastitis, particularly when coupled with the presence of milk deposits, which may provide a nidus for bacterial infections.

Intriguingly, Stat3 also regulates expression of members of the chloride channel regulators, calcium activated, (CLCA) family, of proteins during involution. A positive association is observed between murine CLCA1 and CLCA2 and Stat3 activity, whilst Stat3 negatively regulates murine CLCA5, the murine orthologue of human CLCA2 [125]. The exact functions of CLCA family members within the mammary gland are yet to be determined, but their regulation by Stat3 during the involution period may be pertinent to the pathogenesis of involution-associated mastitis given their postulated modulation of the innate immune response and/or potential activity as signalling molecules [126, 127].

It is important to note that cattle are usually in the final trimester of pregnancy during the dry period, and some dairy goats may be pregnant, depending on the production system [128]. Thus, the involution process may be markedly modulated by what we will term a ‘parallel pregnancy signature’. Although some bovine mammary epithelial cells undergo cell death during bovine mammary involution [129], tissue regression is not notable [130]. This particular aspect of bovine involution is therefore an important species difference which needs to be carefully considered when adopting a One Health viewpoint before making inter-species comparisons.

In spite of the differences in progression of involution in cattle, high levels of serum amyloid A3 expression are also observed in bovine mammary epithelial cells during mid to late involution and in inflammatory states [131] indicating that the inflammatory profile of the bovine involution mammary gland may be similar to rodent models. Experiments in which serum amyloid A3 was infused into the mammary gland via the teat canal suggest that serum amyloid A3 may enhance MMP9 activity and may also reduce *Staphylococcus aureus* infection [132].

Abrupt cessation of lactation in sheep heralds a transient increase in gland cistern volume as measured ultrasonographically. Interestingly, approximately one week after weaning, milk within the gland cistern exhibits ultrasonographic evidence of clotting and is interpreted to be gradually resorbed, resulting in a reduction in gland cistern volume [133]. Again, it is possible that accumulation of milk within the gland cistern may represent a potential nidus for infection, particularly if there is compromise to the innate immune defences of the teat canal such as through mechanical injury. Similar to murine models, the ovine mammary gland also exhibits involution-associated mammary epithelial cell death, efferocytosis mediated by macrophages and mammary epithelial cells, and ultrasonographic evidence of matrix remodelling [59, 133].

### Importance of the Dry Period in Dairy Ruminants

There is a bi-directional relationship between the dry period and mammary health and the dry period represents a time utilised to cure cows or small ruminants from subclinical mammary infections prior to the next lactation cycle, concomitantly improving milk quality [104, 134]. The nonlactating period between drying off and parturition is also an important period of renewal in dairy animals, with the first thirty days of the dry period considered to represent a phase of active involution, and the subsequent thirty days a period of cellular renewal [24].

## Mouse Models of Mastitis

Mouse models of mastitis provide a tractable system in which the mammary microenvironment can be manipulated in a controlled manner and therefore offer a model in which the effects of new interventions can be more readily elucidated [97, 102]. However, some protocols involve weaning of offspring at the time of mastitis induction which causes induction of involution [135]. This may not recapitulate the changes in the breast microenvironment, for example lactating women are often advised to continued breastfeeding where feasible and clinically indicated [136]. In addition, anatomical differences should also be considered. For instance, as discussed above, the mammary stroma varies between humans, ruminants, rabbits and laboratory rodents, and so extrapolation between species requires prudence and an appreciation of such differences. There may be elements of disease pathogenesis in which spontaneous mastitis in large animals more closely recapitulates the condition in humans, and as such study of ruminant mastitis may yield particularly useful insights.

## Concluding Remarks

Clearly the environmental conditions in which humans, dairy ruminants, and rabbits live frequently vary considerably [137] and it has been suggested that this factor may militate against the utility of a One Health approach. The conclusion of this review is certainly not the view that a One Health focus holds the answer to every outstanding question regarding the pathogenesis and treatment of mastitis in humans and animals, and as we have stated throughout, an awareness of species differences, as well as similarities, is of paramount importance. However, as one of many ‘tools in the armoury’ of those involved in mastitis research, maintaining a One Health perspective opens up additional opportunities and possibilities.

Mastitis is a painful condition, and at the heart of any scientific discussion about this condition should be an approach mindful of the potential psychological effect of mastitis on the human mother and infant, and the likely welfare impact on production and laboratory animals. A One Health mindset will foster cross-fertilization of ideas between biomedical scientists, whether trained in basic, medical or veterinary sciences, and may offer the chance to expedite progress in this important field of research.

## Materials and Methods for Unpublished Experiments

### Animals

Rabbits were shot for population management and cadavers were donated to the anatomic pathology service of the Department of Veterinary Medicine, University of Cambridge, for research and teaching purposes. At the time of post mortem examination, rabbits were weighed to assess maturity [138]. Macroscopic post mortem assessment included examination of the mammary glands and reproductive tract. Where appropriate, stage of pregnancy was determined by assessment of foetal crown-rump measurement. Mammary tissue from cattle and sheep was collected from ruminants examined by the anatomic pathology service of the Department of Veterinary Medicine, University of Cambridge, with owner consent for collection of tissues for research purposes.

### Histology and Immunohistochemisty

Mammary tissue was collected in 10% neutral-buffered formalin, and was subsequently processed and sectioned. Staining with haematoxylin and eosin followed standard histological methodology. Immunohistochemical (IHC) staining for CD3 (dilution 1:150; mouse monoclonal, clone F7.2.38, Dako Pathology/Agilent Technologies LDA UK, Cheadle, Cheshire, UK) and CD20 (dilution 1:400; rabbit polyclonal, ThermoFisher Scientific, 168 Third Avenue, Waltham, MA. USA 02451) followed a routine protocol using an automated IHC system (Dako Pathology/Agilent Technologies).

## Abbreviations

CAEV: Caprine arthritis and encephalitis virus
CLCA: Chloride channel regulators, calcium activated
E: Embryonic day
FR: Fürstenberg's rosette
HIV: Human immunodeficiency virus
IHC: Immunohistochemical
LIF: Leukemia inhibitory factor
LPS: Lipopolysaccharide
LTA: Lipoteichoic acid
MMP: Matrix metalloproteinase
MVV: Maedi-visna virus
OPPV: Ovine progressive pneumonia virus
SCM: Subclinical mastitis
SRLV: Small ruminant lentivirus
STAT3: Signal transducer and activator of transcription 3
TLR: Toll-like receptor
